# How Do Price and Quantity Promotions Affect Hedonic Purchases? An ERPs Study

**DOI:** 10.3389/fnins.2019.00526

**Published:** 2019-05-29

**Authors:** Kunpeng Jing, Yupeng Mei, Zhijie Song, Hao Wang, Rui Shi

**Affiliations:** School of Economics and Management, Yanshan University, Qinhuangdao, China

**Keywords:** price promotion, quantity promotion, hedonic purchase, P2, N2, LPP

## Abstract

Due to consuming hedonic products unnecessary to basic well-being, consumers need justifications for pleasure. However, different justifications have differential influences in promoting hedonic purchases, such as price and quantity promotions (PP and QP), the difference being that the latter requires purchasing additional units to get the same discount as the former. In the present study, even-related potentials (ERPs) was applied to reveal the timing of brain activities to further understand how promotion information consisting of promotion type (PP and QP) and discount depth, deep and shallow discounts (DD and SD) on hedonic products was processed. Behaviorally, consumers were more willing to purchase items in PP and DD conditions than QP and SD conditions, respectively, and spent more time making final purchase decisions in QP and DD condition or PP and SD condition compared to PP and DD condition. Neurophysiologically, DD automatically recruited more attentional resources than SD and led to a higher P2 amplitude. QP and DD condition or PP and SD condition evoked a larger N2 amplitude and enhanced perceptual conflict compared to PP and DD condition. During late stage, PP and DD elicited a more positive LPP amplitude in contrast to QP and SD, respectively, indicating that people have stronger purchase intention and positive affect in PP and DD contexts. These findings provided evidence for the differential influences between PP and QP and what ultimately made consumers buy hedonic products or not.

## Introduction

Consumers not only spend a lot of money in utilitarian consumption for basic needs and achievement of goals but also purchase hedonic products for pleasure and to improve living standards in the course of their everyday life. It is noteworthy that hedonic consumption has an inherent disadvantage because of its lack of necessity for basic well-being (Berry, 1994), leading to emotional ambivalence (Ramanathan and Williams, 2007) whereby hedonic products and service can make people relax and allow them to enjoy something while they will afterward feel guilty as a result of acting indulgently.

Therefore, consumers need a good reason to justify their hedonic consumption (Kivetz and Simonson, 2002a,b; Okada, 2005; Lu et al., 2016; Kivetz and Zheng, 2017). For example, when we work hard and have achieved a long-term goal, we may do something fun, such as having dinner at a French restaurant or traveling around the world, to indulge ourselves and improve the quality of life. In other words, we have earned the right of hedonic consumption by effort (Kivetz and Simonson, 2002a). In fact, there are various justifications for acquisition of hedonic goods, like donations to charity (Strahilevitz and Myers, 1998; Zemack-Rugar et al., 2016) and promotions (Wertenbroch, 1998; Khan and Dhar, 2010; Lee-Wingate and Corfman, 2010; Kivetz and Zheng, 2017). Strahilevitz and Myers (1998) treated donation as a justification for purchases, and products were divided into two types, including utilitarian and hedonic goods, and their study suggested that promised donations had a greater effect on promotion of luxuries than of necessities, especially for guilt-sensitive consumers (Zemack-Rugar et al., 2016). Promotions, one of the most popular means to spur demand for products from retailers, can also be regarded as a justification for hedonic consumption. Lee-Wingate and Corfman (2010) found that gifts-with-purchase promotions, whereby buying one item could earn a gift, would increase the likelihood of purchasing hedonic products if the gift was practical. However, different types of promotions have differential influences on hedonic consumption, such as price and quantity promotions (QPs). Kivetz and Zheng (2017) showed that consumers toward a hedonic goal demonstrated a stronger purchasing motivation toward price discount in comparison to the QP condition, while purchasing utilitarian products had an opposite pattern, that is, people expressed a preference for QP. Rational persons will purchase a number of utilitarian products for basic needs in the QP condition even though they cannot use these items at the time of purchase (Frankfurt, 1984). Conversely, consumers are insensitive to the price of hedonic products (Wertenbroch, 1998) such that they have limited demands for hedonic consumption regardless of discount. Consumers never go to great lengths to justify their utilitarian consumption and only consider its benefit and their own basic needs. As the offer of QP is appropriate and effective by this purchase pattern, consumers’ preferences are easily understandable. However, purchasing hedonic products is not the case, for consumers need justification to do it other than the transaction value of products. Price promotion (PP) is a better reason than QP, but it is difficult to understand the insensitivity to price. The phenomenon of the differential impact between price and QPs on hedonic products is explained not clearly. There is little consideration for discount depth staying the same in the hedonic consumption domain. However, discount is an important factor in marketing correlation studies as it has a strong effect on perceived transaction value (Kwon et al., 2010; Cai et al., 2016) and behavioral reactions of consumers (Huang and Yang, 2015) such that there might be discrepancies in purchase incentives when people with hedonic purpose are exposed to different depths of discount. Additionally, it is essential to have insight into consumers’ underlying neural mechanisms closely related to hedonic consumption, since self-reported methods generally adopted by previous studies demonstrate intra-personal aspects less objectively than neuroscientific methods (Kuan et al., 2014), and these aspects include why people are not willing to purchase various hedonic products, how information about price and QPs is processed and what ultimately makes consumers purchase hedonic products or not.

The current study applied event-related potentials (ERPs), a non-invasive technology which has the advantage of excellent time resolution, helping to reveal the timing of brain activities (Friedman and Johnson, 2000), in contrast to other neuroscientific methods to investigate how price and quantity promotions (PP and QP) affect hedonic purchases and whether the processing of information of promotions is modulated by DD and SD.

There are three ERP components associated with cognitive processes and discussed in this present study, including the P2 and N2 components and the late positive potential (LPP). P2 is an early positive potential generally over frontal areas with a peak latency from 100 to 200 ms after the onset of a stimulus (Carretié et al., 2001; Delplanque et al., 2004). Previous studies have suggested that negative stimuli could recruit attention resources more automatically and thus elicited a larger P2 amplitude than positive stimuli (e.g., Carretié et al., 2001; Wang et al., 2012; Jin et al., 2018), which was conducive to rapid reaction to danger stimuli and appropriate decision-making (Yuan et al., 2007). In addition, it is of significance that an enhanced P2 amplitude can also be observed when subjects are exposed to positive rather than neutral stimuli (Herbert et al., 2006; Lin et al., 2015). For example, Herbert et al. (2006) showed that a higher P2 amplitude could be evoked at the time of the evaluation of positive rather than neutral emotional adjectives. In the current study, neither PP nor QP are negative messages for consumers, and as people with a hedonic purpose give an emotional preference to PP, different promotions might reflect the divergence in the P2 component.

N2 is common in ERPs and is a negative component with a frontal-central scalp distribution peaking at around 250–350 ms after stimulus presentation (Folstein and Van Petten, 2008). A considerable number of studies suggested that this component was associated with cognitive conflict such as mismatch (Folstein and Van Petten, 2008; Kemper et al., 2012; Han et al., 2015), cueing conflict (Gajewski et al., 2008; Prime and Jolicoeur, 2009) and perceptual conflict (Ma et al., 2007, 2010; Wang et al., 2016, 2018; Jin et al., 2017). For instance, Kemper et al. (2012) reported the effects of explicit expectation in a match task where participants needed to remember the cue given by the computer or their own prediction for judgment on a consistent one-to-one match between the color or shape of a subsequent stimulus and the cue or prediction, suggesting that mismatches and response matches could elicit an enhanced N2 amplitude in contrast to stimulus matches, especially in the prediction condition. Furthermore, in the neuromarketing domain, Wang et al. (2018) showed that giving five-star reviews for coupons could decrease the conflict between personal interests and immorality, which was manifested in the less negative N2 component compared to monetary reward without any requirement. Given that people want to purchase only a few hedonic products (Wertenbroch, 1998), QP is inconsistent with buying habits of consumers and thus they could detect enhanced perceptual conflict in QP condition in contrast to PP.

LPP is a long latency P300 component over widespread distribution from the frontal to the parietal sites, with a minimum over the frontal area and maximum over the parietal (Cacioppo et al., 1993, 1994), which occurs between 300 and 1200 ms after stimulus presentation. Previous studies demonstrated that the LPP component was associated with motivated attentional processing, which was reflected in arousal and motivation (e.g., Schupp et al., 2000; Gable and Harmon-Jones, 2010; Ma et al., 2018; Wang et al., 2018). Gable and Harmon-Jones (2010) showed that appetitive pictures of things people desired attracted local attention and aroused more positive emotion, and thus a larger LPP amplitude was observed compared to neutral stimuli. Moreover, Ma et al. (2018) indicated the influences of the attribute priming effect on bundling and they found that purchasing a product bundled with another free item could elicit a more positive LPP component, enhanced purchasing motivation and sustained attentional processing compared to other purchases of bundles. Since consumers suggest stronger purchase intent and higher purchase rate (PR) in PP condition than in QP (Kivetz and Zheng, 2017), there could be different LPP amplitudes between PP and QP in the current study.

As has been introduced above, though it was not clear how discount depth as a moderating factor affected the brain activity of consumers, previous relevant conclusions led to the following predictions: firstly, considering that positive stimuli could elicit a higher P2 amplitude than neutral stimuli and people feel better toward PP than QP, PP would recruit more attention resources and evoke a larger P2 amplitude than QP. Secondly, as the idea that with QP people need to purchase additional units to get certain discount has failed to meet the expectation that they only purchase a few hedonic items for pleasure, there would be enhanced perceptual conflict and a more negative N2 amplitude in the QP condition rather than in PP. Finally, people show stronger purchase motivation and demand for hedonic products is more provoked, which would be manifested in a larger LPP amplitude in PP condition compared to in QP.

## Materials and Methods

### Participants

Twenty (12 females) right-handed students^1^ from Yanshan University, whose ages were from 18 to 23 years (mean age = 21.3 ± 1.3) participated in this experiment. All of them were native Chinese speakers and had normal or corrected-to-normal vision without any history of neurological disorders or mental diseases. Written consent was provided in accordance with the Declaration of Helsinki prior to the experiment and the subjects were paid for their participation after the experiment. The experiment was approved by the Internal Review Board of the Laboratory of Cognitive Neuroscience, Yanshan University.

### Experimental Stimuli

Fifty pictures of snacks like chocolate, cookies and popcorn as hedonic products were used in the experiment, which were familiar to consumers and selected from local supermarkets and Taobao.com, one of the largest online retailers, and one pretest. First, we chose snacks that were sold for around 10 yuan (approximately 1.5 dollars) from two different retailers and subsequently these pictures of snacks were adjusted to the same size. In addition, a pretest was conducted. Thirty-six people were provided with the definition of utilitarian and hedonic products and rated each picture of a snack obtained from the first step and presented at random using seven-point hedonic (1 = not at all hedonic, 7 = extremely hedonic, mean ± s.e.m = 4.026 ± 1.416) and utilitarian scales (1 = not at all utilitarian, 7 = extremely utilitarian, mean ± s.e.m = 2.861 ± 1.474). Only snack pictures whose scores on the hedonic scale were significantly higher (*p* < 0.05) than on the utilitarian scale could be considered for use in the current study. With regard to different types of promotions, purchase volume served as an index to distinguish between PP and QP and, specifically, buying five units to get a certain discount was QP and buying one to get the same discount as with QP was PP in the experiment. As for discount depth, 20 or 30% off was seen as a SD and 60 or 70% off was seen as a deep discount (DD).

### Procedures

**FIGURE 1:**
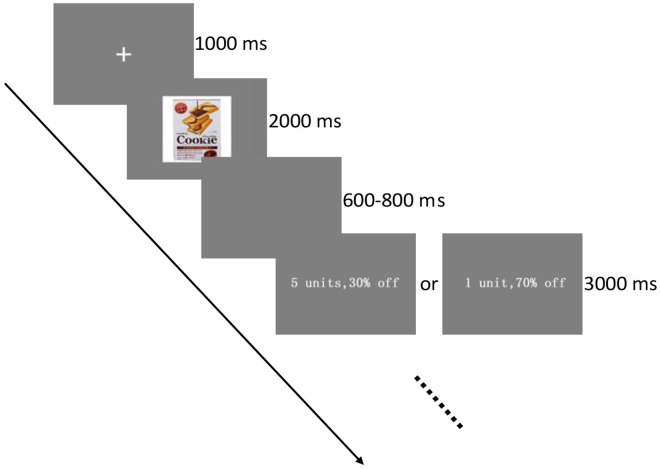
Experimental task: the participants were instructed to make a purchase decision from the promotion information of a certain item.

Subjects sitting on a comfortable chair and maintaining a distance of 70 cm from a 23-inch computer monitor (1,024 × 768 pixels, 60 Hz) completed this task with a visual angle of 2.50 × 2.24 in a sound attenuated room. The Psychophysics Toolbox (Brainard, 1997) was selected to run the program of experiment. As shown in Figure 1, the background color was gray in the course of the experiment. Each trial began with a fixation cross for 1000 ms, followed by a snack picture for 2000 ms. Then, an empty screen was presented for a random amount of time ranging from 600 to 800 ms. At the end of each trial, information on the promotion regarding purchase volume appeared on the right side of a comma located in the center of the background, and the discount depth was displayed on the other side, which then disappeared when participants pressed one of a number of specified buttons on the keyboard or until 3000 ms after the initial presentation. The font used for the information on the promotion was Arial in white, and the discount for each product was identical in DD and SD condition. Half of the participants had to press “f” for “buy” and “j” for “not buy” and the others had an opposite pattern. There were 200 trials in total assigned pseudo-randomly to four blocks, and all promotion conditions for each product did not appear on four consecutive trials of each block.

Before the experiment, the following instructions needed to be simple to participants: first, the original price of every snack that was sealed to maintain freshness enough to finish it regardless of purchase volume was 10 yuan (approximately 1.5 dollars) in this experiment. Second, every time participants were exposed to information regarding a promotion about a kind of snack they had to decide to buy this product or not from a virtual allocation of 80 yuan (around 12 dollars). Additionally, to increase each participant’s motivational engagement, one of the promotion conditions for hedonic products on which the subject had decided to spend money would be randomly chosen for “selling” to him or her, that is, one spent a portion of the RMB 80 (around 12 dollars) allocation to acquire one unit in PP condition or five in QP after the experiment (Knutson et al., 2007; Ma et al., 2018). As a result, one or five units and spare money would be given to the participant. Finally, in order to minimize possible bias induced by buying only a few products and following previous research (Ma et al., 2018), a punitive measure was implemented whereby participants would lose money if the number of promotion conditions in which they decided to buy snacks was less than the minimum. Specifically, if the number of conditions was < 21, 30 yuan would be deducted from the spare money. If the number was between 21 and 25, 15 yuan would be lost and if between 26 and 30, 5 yuan lost. The spare money would not be deducted at all if the number was > 30.

### EEG Recording and Analysis

EEG data was recorded using a Brain actiCHamp amplifier (Brain Products GmbH, Munich, Germany) and a cap containing 64 Ag/AgCl electrodes with a sampling rate of 500 Hz. The amplifier bandpass was 0.05–100 Hz and electrode impedances were kept below 10 kΩ. Cz served as an online reference and electrodes were re-referenced offline to the average of the left and right mastoid references. The vertical EOG and horizontal EOG were recorded from electrodes placed supra- and intra-orbital to both eyes and lateral to the outer canthi of both eyes. ERPs were analyzed by BrainVision Analyzer 2.1 (Brain Products GmbH, Munich, Germany), digitally filtered offline with a low-pass filter at 16 Hz (24 dB/Octave) and segmented into epochs from 200 ms before the onset of the promotion information to 800 ms after onset, with the first 200 ms pre-target interval as a baseline. Trials exceeding ± 100 μV were excluded and ocular artifacts were corrected by the method proposed by Gratton et al. (1983).

ERP recordings were created separately for four experimental conditions (two types of discount depth × 2 types of promotion). According to the visual observation of the grand average waveforms and associated studies mentioned in the introduction, P2, N2, and LPP components were analyzed. Time windows of 160–220 ms, 280–360 ms and 450–600 ms was chosen for analyses of P2, N2, and LPP, respectively. Nine electrodes (F1, Fz, F2, FC1, FCz, FC2, C1, Cz, and C2) over the frontal-central area were included for P2 and N2 and fifteen electrodes (F1, Fz, F2, FC1, FCz, FC2, C1, Cz, C2, CP1, CPz, CP2, P1, Pz, and P2) over the frontal-central-parietal were included for LPP. Repeated-measured analyses of variance (ANOVAs) were performed for these components. The Greenhouse-Geisser correction was used for violation of the sphericity assumption (uncorrected dfs and corrected *p*-values were reported). Spearman correlation analyses were conducted between the N2 amplitude and reaction time (RT) as well as between the LPP amplitude and PR. All values are expressed as mean ± S.E.M.

## Results

### Behavioral Results

**FIGURE 2:**
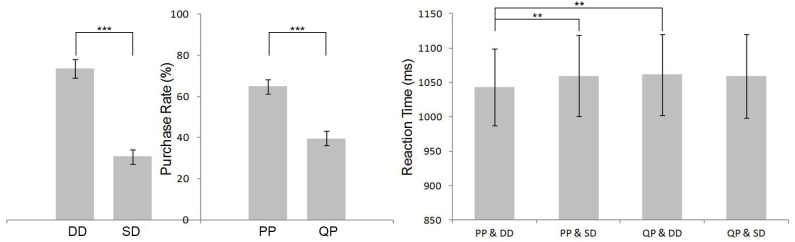
Behavioral results. The purchase rate (PR) (left) and reaction time (RT) (right) for each condition. The error bars suggest standard error of the mean. ^∗∗^*p* < 0.05, ^∗∗∗^*p* < 0.001.

A two-way 2 (promotion: PP vs. QP) × 2 (discount: DD vs. SD) ANOVA was performed for PR and RT. The result of PR is shown in Figure 2. There were significant main effects for promotion (*F*_(1,19)_ = 27.134, *p* < 0.001, $\eta_{p}^{2}$ = 0.588) and discount (*F*_(1,19)_ = 50.144, *p* < 0.001, $\eta_{p}^{2}$ = 0.725) without interaction between the two factors. PR of DD (0.736 ± 0.044) and PP (0.648 ± 0.034) was higher than SD (0.307 ± 0.034) and QP (0.395 ± 0.035), respectively.

As for RT, the ANOVA revealed a significant main effect of promotion (*F*_(1,19)_ = 7.540, *p* < 0.05, $\eta_{p}^{2}$ = 0.284), which was not the case for discount. The RT’s result is shown in Figure 2. The interaction effect between promotion and discount was significant (*F*_(1,19)_ = 10.532, *p* < 0.01, $\eta_{p}^{2}$ = 0.357). Simple effect analyses showed that in the PP condition, RT for SD (1059.648 ± 59.241 ms) was longer than for DD (1042.647 ± 55.963 ms; *p* < 0.01), while in the QP condition there was no significant difference between DD and SD. When the discount was deep, RT for QP (1061.483 ± 58.763 ms) was longer than for PP (1042.647 ± 55.963 ms; *p* < 0.01), while in the SD condition the contrast between PP and QP was not significant.

### ERP Results

**FIGURE 3:**
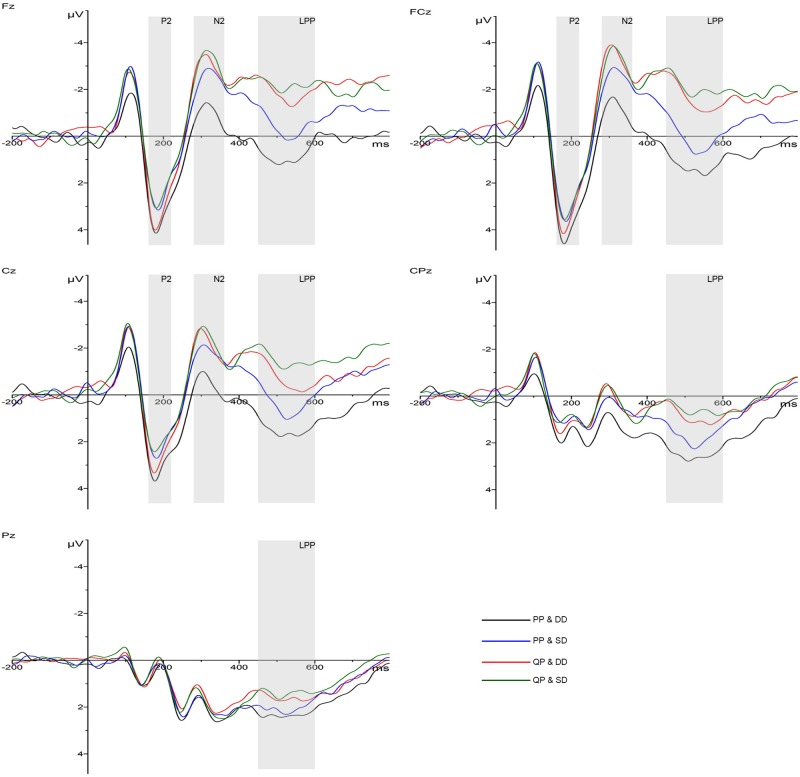
Grand-averaged ERP waveforms from five electrodes: the P2 and N2 amplitudes comparison of the four conditions: PP & DD, PP & SD, QP & DD and QP & SD in three representative electrodes (Fz, FCz, and Cz) and the LPP in five (Fz, FCz, Cz, CPz, and Pz).

The grand-average ERPs are shown in Figure 3. The current source density maps are reported in the Supplementary Material. A three-way 2 (promotion) × 2 (discount) × 9 (electrode) ANOVA was performed for P2 and N2 components. As for P2, there was a significant main effect of discount (*F*_(1,19)_ = 5.202, *p* < 0.05, $\eta_{p}^{2}$ = 0.215). DD (3.286 ± 0.776 μV) elicited a larger P2 amplitude than SD (2.586 ± 0.890 μV). However, there was no significant effect of promotion and interaction effect between them.

With regard to N2, the ANOVA revealed a significant main effect of promotion (*F*_(1,19)_ = 23.805, *p* < 0.001, $\eta_{p}^{2}$ = 0.556), while discount had little effect in this component. The interaction effect between promotion and discount was significant (*F*_(1,19)_ = 11.071, *p* < 0.01, $\eta_{p}^{2}$ = 0.368). Simple effect analyses indicated that in PP condition, SD (-2.318 ± 0.935 μV) elicited a higher N2 amplitude than DD (-0.781 ± 0.760 μV; *p* < 0.05) while in QP condition, there was no significant difference between DD and SD; when in DD condition, a larger N2 amplitude was found in QP (-2.748 ± 0.775 μV) than in PP (-0.781 ± 0.760 μV; *p* < 0.001). While the discount was shallow, the contrast between PP and QP was not significant. Spearman correlation analyses showed that the N2 amplitudes on F2 (*r* = 0.226, *p* < 0.05), FC1 (*r* = 0.236, *p* < 0.05), FCz (*r* = 0.260, *p* < 0.05), FC2 (*r* = 0.244, *p* < 0.05), C1 (*r* = 0.256, *p* < 0.05), Cz (*r* = 0.250, *p* < 0.05) and C2 (*r* = 0.321, *p* < 0.001) were positively related with RT.

A three-way 2 (promotion) × 2 (discount) × 15 (electrode) ANOVA was performed for the LPP component. There were significant main effects of promotion (*F*_(1,19)_ = 34.687, *p* < 0.001, $\eta_{p}^{2}$ = 0.646) and discount (*F*_(1,19)_ = 5.694, *p* < 0.05, $\eta_{p}^{2}$ = 0.231), without interaction between the two factors. PP (1.383 ± 0.709 μV) and DD (0.870 ± 0.685 μV) elicited a higher LPP amplitude than QP (-0.232 ± 0.667 μV) and SD (0.282 ± 0.687 μV), respectively. Spearman correlation analyses showed that the LPP amplitudes on Fz (*r* = 0.248, *p* < 0.05), F2 (*r* = 0.274, *p* < 0.05), FCz (*r* = 0.268, *p* < 0.05), FC2 (*r* = 0.294, *p* < 0.001), Cz (*r* = 0.269, *p* < 0.05), C2 (*r* = 0.283, *p* < 0.05), CPz (*r* = 0.257, *p* < 0.05) and CP2 (*r* = 0.256, *p* < 0.05) were positively related with PR.

All more detailed behavioral and ERP results are reported in the Supplementary Material.

## Discussion

The present study explored how different messages of promotion affected hedonic purchases and were processed at certain times by applying ERPs. Specifically, in this experiment, combining hedonic products with their messages of promotion, participants had to make purchasing decisions in limited time. Additionally, the results of the experiment contributed to developing an awareness of why price and QPs had differential influences on hedonic consumption.

In line with previous studies (Wertenbroch, 1998; Kivetz and Zheng, 2017), PR was higher in PP condition than in QP. Although discount is a temptation to consumers, they do not purchase many discounted hedonic products for pleasure as it is unnecessary for basic needs to spend a lot of money on hedonic consumption, that is, PP can promote purchases of hedonic products effectively. Consumers need PP rather than QP as justification to put an end to the intra-personal conflict between the fact that the purchases are insignificant for their daily life and the need for enjoying themselves (Kivetz and Zheng, 2017). As for discount depth, people suggested stronger purchase intention for hedonic products with a deeper discount. Consumers showed a higher perception of transaction value as deeper discounted products cost less money, which was reflected in a higher PR (Kwon et al., 2010). In other words, according to the justification-based theory (Okada, 2005), discount could become a good reason for justification. As people had a desire to gain bigger profit from this reason, they showed a love of hedonic products in DD condition. The factors of type of promotion and discount depth played important roles in hedonic purchases; however, no significant interaction between the two factors was found.

Previous studies suggested that RT was associated with task difficulty and cognitive load—to be more specific, there is an implication that longer RT infers higher task difficulty (Sweller, 1988; Cowen et al., 2002; Wang et al., 2016). In this present study, there was a significant interaction between promotion and discount. In DD condition, a shorter task completion time for PP than QP was found, suggesting that PP was easy for people to make a final decision while consumers needed to exert extra cognitive effort in QP condition. In the SD condition, there was no difference between PP and QP. Additionally, people could make a response more quickly in the DD condition than the SD condition when type of promotion was PP. Deeply discounted hedonic products made consumers recruit less mental resources for purchase decision than those in the SD condition, leading to an easier task and a shorter RT. In the QP condition, the difference between the DD and SD condition was not significant. However, these results should be treated cautiously as a result of the differential difficulty of calculating a total price across the four conditions.

At the neural level, the P2 component was positively related to attention resources, which reflects an early automatic stage, and is modulated by stimuli of emotional valence (Yuan et al., 2007; Jin et al., 2018). In the current study, only a significant main effect for discount was found. We conjectured that though people felt better in the PP condition than in QP, all promotional messages of hedonic products were positive stimuli and consumers could discriminate valence differences according to discount depth rather than type of promotion at the early stage of processing. The detection of differently positive conditions was more difficult in contrast to negative conditions (Huang and Luo, 2006; Yuan et al., 2007), but sometimes people can distinguish positive and neural stimuli or moderately and extremely positive stimuli such as positive and neutral adjectives (Herbert at al., 2007) and promotion information of DD and SD on the basis of some features from stimuli. Specifically, when exposed to different promotion messages, consumers were independent of conscious inferences and automatically searched a certain attractive aspect of the information. Discount was deemed to be a signal for the allocation of cognitive resources and people preferred DD, which led to a larger P2 amplitude and more attentional resources engaged in the DD condition than in SD. In addition, it is noted that purchase volume, namely, type of promotion was not processed during this phase.

Regarding the N2 component, neuromarketing studies have recently indicated that it was positively related to perceptual conflict (e.g., Jin et al., 2017; Wang et al., 2018). The result on this component showed an interaction of discount and promotion, and there was significant difference between PP and QP in DD condition but not in HD and between DD and SD in PP but not in QP. For hedonic products, participants wanted to spend little money for pleasure, leading to the formation of the implicit expectation that low price or small purchase volume served as standards stemming from the lack of necessity as it relates to basic needs. In the DD condition, QP that required additional purchasing units and money was not up to common buying habits for hedonic products, in other words, it was a mismatch condition and, therefore, higher perception of conflict and a more negative N2 amplitude emerged. Analogously, in the PP condition, DD was consistent with the expectation of participants, and the detection of less cognitive conflict elicited a decreased N2 amplitude. From the justification-based standpoint, all promotion conditions related to the current study could ease the conflict between unimportance of basic needs and pleasure for hedonic consumption. However, the DD and PP conditions alleviated the perceptual conflict more significantly than did the DD and QP condition or the SD and PP condition. Moreover, as N2 is located in the anterior cingulate cortex (ACC) (Yeung and Cohen, 2006) which responds to decision difficulty (Shenhav et al., 2014; Gajewski et al., 2016), N2 is associated with cognitive effort and task difficulty. The higher the N2 amplitude, the more difficult the task, which enables subjects to spend more time in making a final decision. Specifically, the DD and QP condition and the SD and PP condition were more difficult and attracted more cognitive resources to reach the threshold of decision-making compared to DD and PP condition as the result of RT showed.

We obtained significant main effects for discount depth and type of promotion but no interaction between them for the LPP amplitude in this present study during the late cognitive processing stage. As mentioned in the introduction, higher LPP amplitude meant stronger motivation and higher arousing affect (e.g., Schupp et al., 2000; Ma et al., 2018). The stronger the motivation consumers hold, the more they are willing to buy hedonic items and the higher the PR is, indicating that LPP is positively related to PR. Consumers indicated stronger positive affect and purchase intention and thus a larger LPP amplitude in DD and PP conditions than in SD and QP, respectively, as the former were up to the expectation for hedonic consumption. Before making a final purchase decision, there must be perfect excuses for keeping the conflict of spending unnecessary money for well-being to a minimum. DD and PP were seductive enough to lead to the purchase of hedonic items for pleasure compared to SD and QP, respectively, as a result of the insensitivity of price and self-interest, that is, people had stronger motivation to purchase when exposed to promotion messages including the former, which was also supported by PR’s result. As for affect, though both positive and negative stimuli could evoke larger LPP amplitudes compared to neutral stimuli (Olofsson et al., 2008), considering the result of PR, we speculated that as relatively positive information (DD or PP) can lead to higher PR, high arousing positive stimuli enhanced motivated attentional processing as there was positive correction between arousal and motivationally relevant (Bradley, 2009) in this present study. Accordingly, at this stage the two factors, type of promotion and discount depth, were processed.

Generally, three components—P2, N2, and LPP—indicated a three-stage pattern from unconscious to elaborative processes in this study. First, people automatically and rapidly sought out the attractive feature and discount depth, and the deeper the discount, the larger the P2 amplitude. Then, they judged whether the promotion message of a certain hedonic product matched the expectation for low price and purchase volume, and mismatch condition would elicit enhanced perceptual conflict and a larger N2 amplitude. Finally, promotion and discount, respectively, were considered for making a final decision. Specifically, people would have stronger incentives and feel better for higher discount or less hedonic products, leading to higher LPP amplitudes and PRs. A marked difference between the first and third stages was that only discount depth was processed in the former while promotion information was processed completely in the latter, suggesting that people valued outcome (discount depth) rather than process (requirement for purchase volume), which could be efficient for purchase decision as they firstly identified the transaction value of each hedonic product and subsequently whether the purchase volume was appropriate or not. Additionally, in one study by Wertenbroch (1998), subjects were offered the opportunity to buy one and three bags of a new brand of 25% fat (hedonic) and 75% fat-free (utilitarian) potato chips, and when price became increasingly low, the probability of choosing three bags of the former increased less compared to the latter. As a matter of fact, combined with ERP results, the insensitivity of price did not appear at the early stage, in contrast, there was a significant difference in P2 amplitudes between DD and SD and no difference between PP and QP. People expressed a preference for DD unconsciously whereas during the late elaborative stage the consideration of purchase volume and discount led the insensitivity.

However, we acknowledge some limitations of this study. First, the difficulty of calculating the total price for all the promotion information had an impact on behavioral and neural results. PP might be more difficult than QP for calculating total price as the former required the subjects to purchase only one item and there might be differential difficulty for different discount depth, which partially influences PR, RT and ERP components as difficulty is associated with cognitive demand. Moreover, in the SD condition, there was no difference between PP and QP in RT and N2, which might be mainly because of the learning effect. We conjectured that only the optimal selection, the PP and DD condition, was seen to explain the expectation for goods as time went on and thus all other conditions in which people needed to exert extra cognitive effort enhanced perceptual conflict. Moreover, when consumers do shopping, one of the basic rules is their preference for some products, an important factor of marketing. Because studies hedonic consumption rarely involving this factor, especially in the neuromarketing domain, future research should explore past experience with hedonic products or service, personality factors, cultural background and so on.

## Conclusion

The current study explored how PP and QP influenced hedonic purchases at the neural level. Considering discount depth as a moderating factor, less conflict and stronger motivation were detected, reflected in decreased N2 amplitude corresponding to RT related to task difficulty and larger LPP leading to higher PR in the PP condition than in QP when discount was deep. Meanwhile, only enhanced LPP was elicited by the PP condition in comparison to QP in the SD condition. Moreover, it was easier for discount depth to distinguish stimuli rapidly compared to purchase volume, and DD was more attractive and evoked larger P2 and LPP amplitudes than SD. These findings indicated that promotion information could be processed not entirely at different times and only one factor, discount depth, played a role in P2 amplitude. Meanwhile, the N2 and LPP components were affected by discount depth and type of promotion, which could lead to better understanding of the differential influences on hedonic products and facilitate the study on justification-based theory and hedonic consumption.

## Ethics Statement

This study was carried out in accordance with the recommendations of Declaration of Helsinki, the Internal Review Board of the Laboratory of Cognitive Neuroscience, Yanshan University, with written informed consent from all subjects. All subjects gave written informed consent in accordance with the Declaration of Helsinki. The protocol was approved by the Internal Review Board of the Laboratory of Cognitive Neuroscience, Yanshan University.

## Author Contributions

KJ, YM, ZS, and HW conceived and designed the experiment. KJ, YM, and ZS performed the experiment. KJ and HW analyzed the data. KJ, YM, and RS wrote and edited the manuscript.

## Conflict of Interest Statement

The authors declare that the research was conducted in the absence of any commercial or financial relationships that could be construed as a potential conflict of interest.

## Footnotes

1 Sample sizes in 13 to 28 ranges are suitable for ERP studies involving multiple trials and posterior analyses (Wang et al., 2016).
