# Supplementary material for: How Do Price and Quantity Promotions Affect Hedonic Purchases? An ERPs Study
Source: Front Neurosci. 2019 May 29;13:526. doi: 10.3389/fnins.2019.00526 (PMC6558398; doi:10.3389/fnins.2019.00526)
Supplement: Supplementary file 1 [file Data_Sheet_1.pdf]

		Discount		Sum	F (p) (η_p^2)
		DD	SD		
		Mean (SE)	Mean (SE)		
Promotion	PP	0.841 (0.035)	0.454 (0.056)	0.648 (0.034)	27.134 (0.000) (0.588)
	QP	0.630 (0.062)	0.160 (0.030)	0.395 (0.035)	
Sum		0.736 (0.044)	0.307 (0.034)	Interaction effect: F = 3.146, p = 0.092, η_p^2 = 0.142	
F (p) (η_p^2)		50.144 (0.000) (0.725)			

Supplementary Table 1. The ANOVA's result of purchase rate (PR), including mean values across four conditions (PP & DD, PP & SD, QP & DD and QP & SD), standard error of the mean (SE) and main and interaction effects.

		Discount		Sum	F (p) (η_p^2)
		DD	SD		
		Mean (SE)	Mean (SE)		
Promotion	PP	1042.647 (55.963)	1059.648 (59.241)	1051.148 (57.553)	7.540 (0.013) (0.284)
	QP	1061.483 (58.763)	1058.956 (60.820)	1060.219 (59.768)	
Sum		1052.065 (57.317)	1059.302 (60.103)	Interaction effect: F = 10.532, p = 0.004, η_p^2 = 0.357	
F (p) (η_p^2)		3.413 (0.080) (0.152)			

Supplementary Table 2a. The ANOVA's result of Reaction Time (RT), including mean values across four conditions (PP & DD, PP & SD, QP & DD and QP & SD), standard error of the mean (SE) and main and interaction effects.

Condition			Mean Difference (Former – Latter)	SE	p
PP	DD	SD	-17.001	5.772	0.008
	SD	DD	17.001	5.772	0.008
QP	DD	SD	2.527	3.934	0.528
	SD	DD	-2.527	3.934	0.528
DD	PP	QP	-18.836	5.373	0.002
	QP	PP	18.836	5.373	0.002
SD	PP	QP	0.692	3.327	0.837
	QP	PP	-0.692	3.327	0.837

Supplementary Table 2b. The Simple effect analyses' result of Reaction Time (RT), including mean difference values across four conditions (PP & DD, PP & SD, QP & DD and QP & SD), standard error of the mean and p values.

		Discount		Sum	F (p) (η_p^2)
		DD	SD		
		Mean (SE)	Mean (SE)		
Promotion	PP	3.467 (0.729)	2.606 (0.972)	3.036 (0.829)	0.786 (0.386) (0.040)
	QP	3.105 (0.845)	2.566 (0.848)	2.836 (0.828)	
Sum		3.286 (0.776)	2.586 (0.890)	Interaction effect: F = 0.369, p = 0.551, η_p^2 = 0.019	
F (p) (η_p^2)		5.202 (0.034) (0.215)			

Supplementary Table 3. The ANOVA's result of P2, including mean values across four conditions (PP & DD, PP & SD, QP & DD and QP & SD), standard error of the mean (SE) and main and interaction effects.

		Discount		Sum	F (p) (η_p^2)
		DD	SD		
		Mean (SE)	Mean (SE)		
Promotion	PP	-0.781 (0.760)	-2.318 (0.935)	-1.549 (0.808)	23.805 (0.000) (0.556)
	QP	-2.748 (0.775)	-2.639 (0.856)	-2.693 (0.795)	
Sum		-1.764 (0.745)	-2.478 (0.883)	Interaction effect: F = 11.071, p = 0.004, η_p^2 = 0.368	
F (p) (η_p^2)		3.289 (0.086) (0.148)			

Supplementary Table 4a. The ANOVA's result of N2, including mean values across four conditions (PP & DD, PP & SD, QP & DD and QP & SD), standard error of the mean (SE) and main and interaction effects.

Condition			Mean Difference (Former – Latter)	SE	p
PP	DD	SD	1.537	0.541	0.010
	SD	DD	-1.537	0.541	0.010
QP	DD	SD	-0.109	0.373	0.773
	SD	DD	0.109	0.373	0.773
DD	PP	QP	1.967	0.366	0.000
	QP	PP	-1.967	0.366	0.000
SD	PP	QP	0.321	0.314	0.320
	QP	PP	-0.321	0.314	0.320

Supplementary Table 4b. The Simple effect analyses' result of N2, including mean difference values across four conditions (PP & DD, PP & SD, QP & DD and QP & SD), standard error of the mean and p values.

		Discount		Sum	F (p) (η_p^2)
		DD	SD		
		Mean (SE)	Mean (SE)		
Promotion	PP	1.722 (0.751)	0.901 (0.807)	1.311 (0.744)	34.786 (0.000) (0.659)
	QP	-0.107 (0.737)	-0.611 (0.660)	-0.359 (0.690)	
Sum		0.807 (0.719)	0.145 (0.710)	Interaction effect: F = 0.342, p = 0.566, η_p^2 = 0.019	
F (p) (η_p^2)		7.162 (0.015) (0.285)			

Supplementary Table 5. The ANOVA's result of LPP, including mean values across four conditions (PP & DD, PP & SD, QP & DD and QP & SD), standard error of the mean (SE) and main and interaction effects.
