# Supplementary figures and images for: How Do Price and Quantity Promotions Affect Hedonic Purchases? An ERPs Study
Source: Front Neurosci. 2019 May 29;13:526. doi: 10.3389/fnins.2019.00526 (PMC6558398; doi:10.3389/fnins.2019.00526)

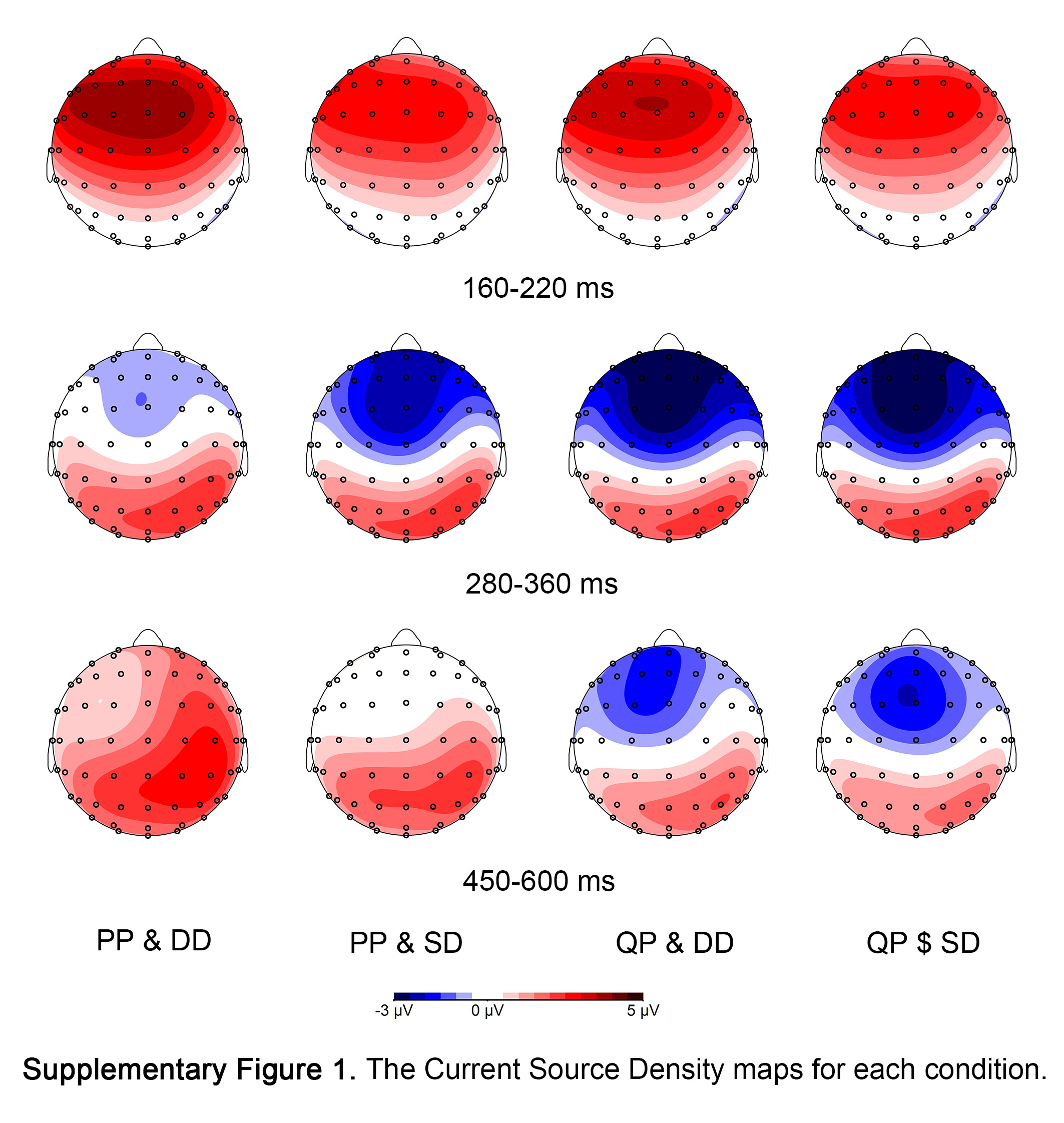

Supplement: Supplementary file 2 [file Image_1.jpg]
